# Searching for the neural correlates of emotional intelligence: a systematic review

**DOI:** 10.7717/peerj.20539

**Published:** 2026-01-08

**Authors:** Víctor Martín-Aguiar, Pablo Fernández-Berrocal, Alberto Megías-Robles

**Affiliations:** Department of Basic Psychology, University of Málaga, Malaga, Spain

**Keywords:** Emotional intelligence, Systematic review, Neural correlates, Brain, MRI, fMRI, EEG

## Abstract

The concept of emotional intelligence (EI) has gained significant interest in the scientific community in recent years. Despite its demonstrated impact on social and personal functioning, the neural bases underlying EI remain poorly understood. This study aimed to conduct a comprehensive systematic review of the existing literature on the neural correlates of EI. The search was conducted in Web of Science, Scopus, PsycINFO, and PubMed databases. A total of 849 studies were initially identified (after duplicates were removed), of which 34 met the inclusion criteria and were selected for the final synthesis. These studies employed various brain mapping techniques, including lesion studies, grey and white matter structural magnetic resonance imaging (MRI), task-based functional magnetic resonance imaging (fMRI), resting-state fMRI, and electroencephalogram (EEG). The findings of this review suggest that EI is supported by a complex and widespread brain network primarily implicated in the integration of cognitive and emotional processes, with significant involvement of structures commonly linked to social cognition. The literature mainly emphasized the role of the insula, ventromedial prefrontal cortex, orbitofrontal cortex, cingulate cortex, and amygdala in conjunction with brain networks comprising these areas, such as the somatic marker circuitry and the social cognition network. Other brain regions, including the dorsolateral prefrontal cortex, cuneus, precuneus, fusiform gyrus, superior temporal gyrus, cerebellum, parahippocampal gyrus, inferior frontal gyrus, frontopolar gyrus, superior parietal lobule, and superior longitudinal fasciculus (SLF) were also frequently mentioned. However, further research is needed to clarify the roles of some of these regions in EI. Limitations and future lines of research are discussed.

## Introduction

Over the past three decades, emotional intelligence (EI) has attracted significant attention in scientific research. To date, a search of the term “emotional intelligence” in the Web of Science (WoS, Clarivate Analytics) databases has identified 15,463 articles featuring this term in the abstract, title, or indexing. Of particular note is the prominence acquired by EI in organizational and educational contexts. However, despite its popularity and recognized importance within research, institutions, and society, the neural mechanisms underlying EI are still poorly understood. This study aims to address this gap by conducting a systematic review of the neurobiological correlates of EI, covering the literature from the inception of the construct through February 2024.

EI was initially conceptualized by Salovey & Mayer (1990). This construct is defined as “the ability to perceive accurately, appraise, and express emotion; the ability to access and/or generate feelings when they facilitate thought; the ability to understand emotion and emotional knowledge; and the ability to regulate emotions to promote emotional and intellectual growth” (Mayer & Salovey, 1997, p. 10).

It is important to note that various approaches to EI exist in the literature. Joseph & Newman (2010) proposed a classification based on the theoretical conceptualization of EI (ability or mixed EI) and the type of measurement tool (self-report or performance-based). They identify three models:

(1) The performance-based ability model understands EI as a mental ability involving emotional perception, facilitation, understanding, and management. It is based on the set of emotional abilities included in the EI conceptualization proposed by Mayer & Salovey (1997). In this model, EI is assessed objectively through performance-based tests in which individuals must solve problems that have correct and incorrect answers. An example of a measurement instrument is the widely used Mayer-Salovey-Caruso Emotional Intelligence Test (MSCEIT; Mayer, Salovey & Caruso, 2002).
(2) The self-report ability model shares its theoretical foundation with the performance-based ability model. However, although it also considers EI as a set of emotion-related abilities, it relies on self-report instruments in which individuals estimate their own EI abilities. The three most representative examples of self-report ability measures are the Schutte Emotional Intelligence Scale (SEIS; Schutte et al., 1998), the Trait Meta-Mood Scale (TMMS; Salovey et al., 1995), and the Wong and Law Emotional Intelligence Scale (WLEIS; Wong & Law, 2002).
(3) The self-report mixed model, unlike the ability models, conceptualizes EI as a broader construct that encompasses both emotion-related mental abilities and personal competencies such as empathy, motivation, impulse control, stress tolerance, flexibility, and other personality factors (Bar-On, 2004). This model uses self-reports, with the most popular instruments being the Bar-On Emotional Quotient Inventory (EQ-i; Bar-On, 2004) and the Trait Emotional Intelligence Questionnaire (TEIQue; Petrides & Furnham, 2009).

From the definitions of each model, substantial theoretical and methodological differences emerge. Research has shown weak convergent validity not only between ability and mixed models, but also between the two ability models, with correlations ranging from low to moderate, suggesting that they assess related but distinct aspects of EI (Brackett & Mayer, 2003; Goldenberg, Matheson & Mantler, 2006; Joseph & Newman, 2010; Webb et al., 2013). Moreover, the literature reports discrepant findings regarding the prediction of behavior. Performance-based ability models have demonstrated greater predictive validity in cognitive tasks with emotional content than self-report EI instruments (Gutiérrez-Cobo, Cabello & Fernández-Berrocal, 2016, 2017). In contrast, predictive validity in clinical, social, academic, and occupational contexts has yielded mixed results, with either model showing superior predictive value depending on the criterion variable and the specific study examined (Brackett et al., 2006; Brackett & Mayer, 2003; Denogent et al., 2025; Gómez-Leal et al., 2018; Gong & Jiao, 2019; Joseph & Newman, 2010). The primary limitation of the mixed model stems from its lower theoretical robustness and reduced scientific rigor, as it attempts to integrate a broad spectrum of factors that are difficult to reconcile within a single construct (Joseph & Newman, 2010; Mayer, Salovey & Caruso, 2000; Mayer, Caruso & Salovey, 2016). Indeed, mixed models show a notable overlap with personality measures, whereas this overlap is minimal or absent in performance-based ability models (Van Zyl & de Bruin, 2012; O’Connor & Little, 2003; Webb et al., 2013). Finally, models that rely on self-reports tend to produce more subjective responses and are more susceptible to social desirability bias than performance-based measures (Brackett et al., 2006; Goldenberg, Matheson & Mantler, 2006; Webb et al., 2013). In summary, when studying EI, it is essential to consider the unique characteristics and limitations of each model.

Throughout the literature, higher EI has consistently been associated with multiple positive outcomes across different domains. These include better social relationships and interactions with others (Lopes et al., 2004, 2005), enhanced academic and professional success (Brackett, Rivers & Salovey, 2011; MacCann et al., 2020; Miao, Humphrey & Qian, 2017; O’Boyle et al., 2011), a lower tendency to engage in aggressive, antisocial, and risk behaviors (Megías et al., 2018; Megías-Robles, Sánchez-López & Fernandez-Berrocal, 2022; Sánchez-López et al., 2022; Vega et al., 2022), a reduced likelihood of substance use (Kun & Demetrovics, 2010; Megías-Robles, Perea-Baena & Fernández-Berrocal, 2020), protective effects against depression and suicidal behavior (Cha & Nock, 2009; Downey et al., 2008; Domínguez-García & Fernández-Berrocal, 2018), and greater well-being and life satisfaction (Fernández-Berrocal & Extremera, 2016; Llamas-Díaz et al., 2022).

Despite the extensive body of literature on EI and its social and scientific relevance across numerous domains, the neural bases of EI remain unclear. Existing reviews on this topic are scarce, somewhat outdated (Raz & Zysberg, 2015), or focused on specific brain analysis techniques (*e.g*., lesion studies in Hogeveen, Salvi & Grafman, 2016). Given the importance of EI and the limitations of previous reviews, we consider it essential to advance knowledge of its neural basis through a more up-to-date and comprehensive review of the literature.

To this end, we were interested in covering a broad range of neuroimaging techniques (magnetic resonance imaging (MRI), functional magnetic resonance imaging (fMRI), and electroencephalogram (EEG), among others), as each provides unique and complementary insights into cerebral anatomy and function. For example, structural MRI provides high-resolution anatomical data, enabling the identification of regional gray and white matter (GM and WM) correlates of EI; however, it does not provide information about neural activity (Turner, 2016; Yousaf, Dervenoulas & Politis, 2018). fMRI leverages blood oxygenation level-dependent (BOLD) contrast to map functional activation, excelling in spatial resolution and allowing the mapping of activation patterns and connectivity within cortical and subcortical networks underlying emotional and cognitive processes. However, it is limited by its indirect assessment of brain activity and comparatively poor temporal precision (Holdsworth & Bammer, 2008). In contrast, EEG provides millisecond-level temporal precision, making it ideal for capturing rapid brain dynamics and oscillatory patterns associated with emotional processing. While EEG excels in temporal resolution, its spatial resolution is limited compared to MRI and fMRI (Babiloni et al., 2009). The integration of these approaches offers a richer and more integrative account of the brain’s organization and functioning.

Drawing on the considerations discussed above, this study aimed to conduct a comprehensive systematic review of the existing research on the neural correlates of EI, encompassing publications from 1990, when EI was first formally introduced (Salovey & Mayer, 1990), to the present. We sought to integrate findings across diverse brain mapping techniques and examine the different conceptual models of EI that have been investigated. This approach not only allowed us to summarize existing evidence but also to identify patterns, highlight inconsistencies, and advance our understanding of the neural mechanisms underlying EI. This manuscript is the peer-reviewed version of a previously published preprint (Martín-Aguiar, Fernández-Berrocal & Megías-Robles, 2024).

## Method

### Search strategy

We searched the literature using four primary electronic databases: Web of Science, Scopus, PsycINFO, and PubMed. The search was restricted to articles published between 1990 and February 2024. To maximize the search results and capture all relevant articles, the term “Emotional intelligence” was combined with the following key terms: (1) Neuroimage, (2) Brain Bases, (3) Neuroscience, (4) MRI, (5) Positron Emission Tomography, (6) fMRI, (7) ERP, (8) EEG, (9) Neurobiology, (10) Neural Bases, (11) Neural correlates, and (12) Brain correlates. An example of the Boolean syntax used to search PubMed is provided below: (“Emotional intelligence”) AND (“Neuroimage” OR “Brain Bases” OR “Neuroscience” OR “MRI" OR “Positron Emission Tomography” OR “fMRI” OR “ERP” OR “EEG” OR “Neurobiology” OR “Neural Bases” OR “Neural correlates” OR “Brain correlates”).

Two authors (V.M.A. and A.M.R.) independently searched and reviewed the selected studies according to the established inclusion and exclusion criteria. Discrepancies were resolved by consensus and discussed with a third author (P.F.B.). The search and selection of articles followed the Cochrane and PRISMA guidelines (Higgins et al., 2024; Page et al., 2021). The PRISMA checklist is provided in the Supplemental Material.

### Inclusion and exclusion criteria

The studies considered in this systematic review met the following inclusion criteria: (1) empirical articles, (2) written in English or Spanish, (3) published in a peer-reviewed journal, (4) employing a validated and standardized EI measurement, and (5) including neuroimaging techniques to study the brain basis of EI. Exclusion criteria were: (1) theoretical or review articles, (2) studies using EI questionnaires but only assessing some individual emotional dimensions rather than the whole EI construct, (3) studies not aimed at identifying specific brain regions (*e.g*., EEG studies without brain source localization), and (4) studies including clinical samples with psychological or brain disorders (*e.g*., schizophrenia, depression, or epilepsy).

### Identification

**Figure 1:**
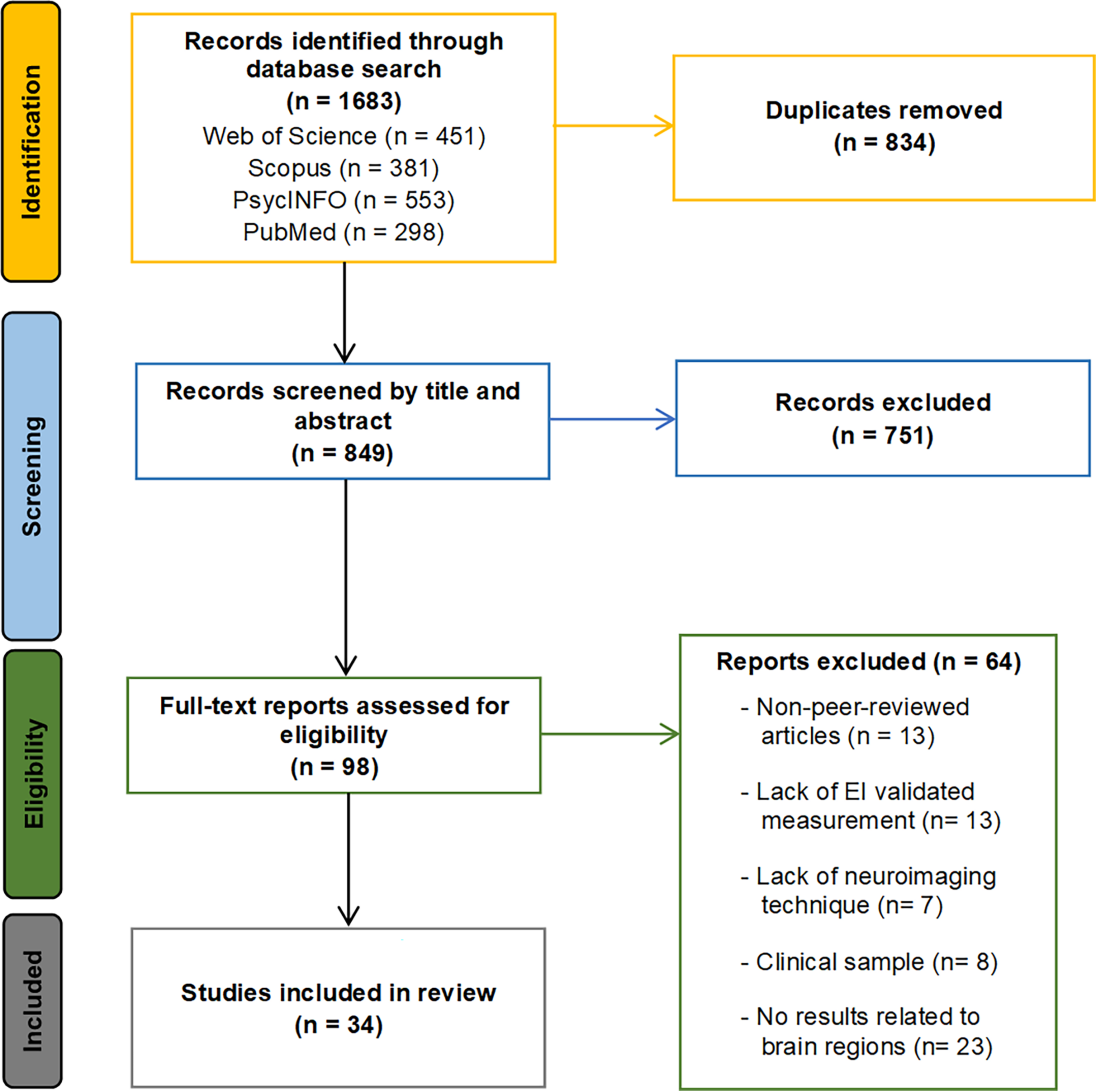
Search and study selection flowchart. The flowchart illustrates the identification phase, with the number of records retrieved from each database; the screening phase; the eligibility phase, including the number of articles excluded for each criterion; and the inclusion phase, showing the final number of studies included in the systematic review.

The initial search across the four databases identified a total of 1,683 articles (451 from Web of Science, 381 from Scopus, 553 from PsycINFO, and 298 from PubMed). After removing duplicates, 849 articles remained. An initial title and abstract screening eliminated 751 articles that were either outside the scope of this review or did not meet the inclusion/exclusion criteria. The remaining 98 articles were considered for full-text review. Sixty-four articles were excluded from this selection for the following reasons: not peer-reviewed journal articles (13 articles), lack of validated EI measures or focusing only on specific EI dimensions (13 articles), absence of neuroimaging techniques (seven articles), failure to report findings related to brain regions (23 articles), and inclusion of clinical sample with psychological disorders (eight articles). Consequently, 34 articles fully met all the established criteria and were included in the systematic review. During the article selection process, inter-rater reliability between reviewers was excellent, with a Cohen’s kappa of 0.91, reflecting a very high level of agreement. Any discrepancies in articles for which there was no clear consensus were resolved by a third reviewer. The literature search and study selection process is summarized in the flow chart presented in Fig. 1.

## Results and description of the identified articles

The studies identified in the systematic review employed a diverse range of neuroimaging techniques, using different assessment and analytical methodologies. To present a clear and organized overview, this section is structured according to the type of neuroimaging technique used. We begin by outlining the key aspects of each study, followed by a brief integrated discussion of the main findings. The characteristics and primary outcomes of all 34 articles are summarized in Table 1. This section concludes with a subsection dedicated to the assessment of study quality.

**Table 1:** Characteristics and main findings of the selected articles (organized according to neuroimaging technique and in alphabetical order of first author’s surname).

Study: First author (year)	Sample size (gender; mean age)	Type of population	Neuroimaging technique	EI instrument (model)	Main findings
Barbey, Colom & Grafman (2014)	152 (152 men; 58.1 years)	American combat veterans (VHIS)	Lesion studies	MSCEIT (performance-based ability model)	Lower EI levels were related to lesions in the right OFC, left inferior and superior parietal cortex, perisylvian language network, dlPFC, temporo-parietal junction, posterior temporal cortex, and in the superior longitudinal, arcuate, superior fronto-occipital, and uncinate fasciculi.
Bar-On et al. (2003)	12 with damage in the SMH circuitry (7 men; 43.5 years)	Patients with brain damage from a Registry of the University of Iowa	Lesion studies	EQ-i (self-report mixed model)	Patients with lesions in the SMH circuitry (vmPFC, amygdala, and insula) showed lower levels of EI.
	12 with brain damage outside the SMH circuitry (4 men; 47.1 years)				
Krueger et al. (2009)	17 with dlPFC lesions (17 men; 57.3 years)	American combat veterans (VHIS)	Lesion studies	MSCEIT (performance-based ability model)	Lower levels in the strategic area of EI were related to vmPFC lesions, while in the experiential area, lower levels were related to dlPFC lesions.
	21 with vmPFC lesions (21 men; 57.9 years)				
	29 with no brain lesions (29 men; 58.3 years)				
Operskalski et al. (2015)	130 (130 men; 59.0 years)	American combat veterans (VHIS)	Lesion studies	MSCEIT (performance-based ability model)	Lower levels of both perceiving and managing EI branches were related to damage in the left temporal pole, left superior temporal gyrus, left insular cortex, and left superior longitudinal fasciculus.
					The perceiving branch was specifically related to damage in the left medial ventral temporal cortex, left fusiform gyrus, right middle frontal gyrus, and left uncinate fasciculus.
					The managing branch was specifically related to damage in the right OFC, right temporal pole, left posterior temporal lobe, left posterior hippocampus, left angular and supramarginal gyri, and left inferior longitudinal fasciculus.
He et al. (2018)	213 (103 men; 20.0 years)	University students	Structural MRI: GM (VBM)	SEIS (self-report ability model)	EI levels were positively related to the right OFC volume.
Killgore et al. (2012)	36 (20 men; 30.0 years)	General population	Structural MRI: GM (VBM)	EQ-i (self-reported mixed model)	The stress management subscale of the EQ-i was positively related to vmPFC volume.
				MSCEIT (performance-based ability model)	MSCEIT total scores were positively related to insula volume and the strategic area was positively related to insula and vmPFC volume.
Koven et al. (2011)	30 (14 men; 30.0 years)	General population	Structural MRI: GM (VBM)	TMMS (self-report ability model)	The attention to emotions subscale was positively related to GM volume in the superior, middle, ventromedial and inferior frontal gyri (mostly right lateralized), bilateral OFC, right frontopolar gyrus, right precentral gyrus, bilateral dorsal anterior CC, and right cuneus.
					The emotional repair subscale was positively related to GM volume in the left anterior CC and right supramarginal gyrus.
Li et al. (2021)	65 (32 men; 37.3 years)	General population	Structural MRI: GM (Morphological Network Efficiency)	SEIS (self-report ability model)	EI scores were positively related to the global efficiency of the morphological brain network in middle-aged participants, while this relationship was negative in young adults.
					EI scores for middle-aged participants were positively related to nodal efficiency in the sensory motor area, temporal pole, inferior parietal sulcus, and mainly in regions associated with the DMN.
Takeuchi et al. (2011)	55 (42 men; 21.7 years)	University students	Structural MRI: GM (VBM)	The Japanese version of the EI scale (self-report mixed model)	The intrapersonal factor of EI was negatively related to GM density in the right anterior insula, right cerebellum, cuneus, precuneus, and a cluster extending from the left medial prefrontal cortex to the left lateral frontopolar cortex.
					The interpersonal factor was positively related to GM density in the right superior temporal sulcus.
					The situation management factor was negatively related to GM density in the bilateral vmPFC and positively in the caudal part of the right posterior parietal cortex and the right middle occipital lobe.
Tan et al. (2014)	328 (155 men; 20.0 years)	University students	Structural MRI: GM (VBM)	SEIS (self-report ability model)	The dimension of monitor of emotions was positively related to GM volume in the left insula, right OFC, and left superior parietal lobe and negatively in the left cuneus.
					The dimension of utilization of emotions was positively related to the GM volume in the right parahippocampal gyrus and negatively in the right fusiform gyrus, left middle temporal gyrus, and left superior temporal gyrus.
					The dimension of social ability was positively related to cerebellum volume.
Xiang et al. (2017)	73 (32 men; 21.5 years)	University students	Structural MRI: GM (VBM)	WLEIS (self-report ability mode)	Levels of EI were negatively related to dlPFC volume.
Pisner et al. (2017)	32 (16 men; 29.2 years)	General population	Structural MRI: WM (FA)	MSCEIT (performance-based ability model)	The EI total score was positively related to FA in the left superior longitudinal fasciculus and dorsal corticospinal tract.
					The managing branch of EI was positively related to FA in the *corpus* callosum, cingulum bundle, and uncinate fasciculus.
Takeuchi et al. (2013a)	118 (74 men; 21.6 years)	University students	Structural MRI: WM (FA)	The Japanese version of the EI scale (self-report mixed model)	The intrapersonal factor of EI was positively related to FA in the right anterior insula.
					The interpersonal factor was positively related to FA in the inferior longitudinal fasciculus.
Takeuchi et al. (2019)	1,207 (693 men; 20.8 years)	University students	Structural MRI: WM (MD)	The Japanese version of the EI scale (self-report mixed model)	The intrapersonal factor of EI was negatively related to MD in the right putamen, globus pallidum, posterior insula, and fusiform gyrus and positively related in the medial PFC, anterior CC and left inferior frontal gyrus.
					The interpersonal factor was positively related to MD in the precuneus.
					The management factor was positively related to MD in the anterior CC, insula, and lateral prefrontal cortex.
Alkozei & Killgore (2015)	44 (21 men; 30.4 years)	General population	Task-based fMRI	MSCEIT (performance-based ability model)	EI levels were negatively related to activation in the left posterior insula during the presentation of subliminal angry faces.
Deak et al. (2022)	40 (19 men; 21.0 years)	Not specified	Task-based fMRI	SEIS (self-report ability model)	EI levels were positively related to activation in the hippocampus, parahippocampal gyrus, posterior CC, insula, and superior temporal lobe during the presentation of negative content.
					EI levels were negatively related to activation in the medial frontal gyrus, temporal areas, and cingulate gyrus and positively related in the superior parietal lobe and vision-related regions during the reappraisal process.
Dobrushina et al. (2020)	30 (0 men; 51.0 years)	General population (women only)	Task-based fMRI	MSCEIT (performance-based ability model)	EI levels were positively related to activation in the right anterior insula during the interoceptive task.
Hansenne et al. (2014)	18 in the control group (0 men; 21.0 years) 18 in the experimental group (EI training) (0 men; 21.0 years)	General population (women only)	Task-based fMRI	TEIQue-SF (self-report mixed model)	Using an emotional regulation task, an experimental group trained in EI, compared with a control group, showed lower activation in the bilateral inferior parietal lobe, right precentral gyrus, and intraparietal sulcus when decreasing negative emotions and in the right middle frontal gyrus, left OFC and right frontopolar cortex when increasing positive emotions.
Karle et al. (2018)	85 (42 men; 25.4 years)	Not specified	Task-based fMRI	MSCEIT (performance-based ability model)	EI scores were positively related to the neural response associated with voice-sensitivity in the left amygdala, left anterior insula, and left inferior frontal gyrus.
			Structural MRI: GM (VBM)		EI scores were negatively related to the neural response associated with face-sensitivity in the right fusiform face area.
					EI scores were positively related to GM volume in the right OFC and left insula and negatively in the right fusiform face area.
Killgore & Yurgelun-Todd (2007)	16 (9 men; 11.6 years)	Children and adolescents	Task-based fMRI	EQ-i:YV (self-reported mixed model)	EI levels were negatively related to activation in the vmPFC, amygdala, insula, middle cingulate gyrus, hippocampus, and parahippocampal gyrus and positively related to activation in the left cerebellum and right occipital cortex during the presentation of fearful faces.
Killgore et al. (2013)	39 (22 men; 29.9 years)	General population	Task-based fMRI	EQ-i (self-reported mixed model)	EQ-I scores were not related to any change in brain activation.
				MSCEIT (performance-based ability model)	MSCEIT total scores were positively related to activation in the vmPFC and rostral anterior CC when facial expressions decreased in trustworthiness.
Kreifelts et al. (2010)	24 (12 men; 26.6 years)	General population	Task-based fMRI	SEIS (self-report ability model)	EI scores were positively related to activation in the right posterior superior temporal sulcus when performing a task involving audiovisual integration of emotional signals.
Quarto et al. (2016)	63 (29 men; 29.4 years)	General population	Task-based fMRI	MSCEIT (performance-based ability model)	EI scores were positively related to activation in the left insula for fearful faces and negatively for angry faces when performing a social judgment task.
Reis et al. (2007)	16 (6 men; 21.7 years)	University students	Task-based fMRI	MSCEIT (performance-based ability model)	EI levels were negatively related to activation in the left anterior temporal and left frontopolar regions during a social reasoning task.
Bajaj & Killgore (2021)	55 (26 men; 30.1 years)	General population	Resting-state fMRI (effective brain connectivity)	MSCEIT (performance-based ability model)	EI total scores were not related to changes in effective brain connectivity.
					The facilitating and managing branches were related to changes in effective brain connectivity in regions participating in the control-execution network.
					The understanding branch was related to changes in effective brain connectivity in regions participating in the salience network.
Killgore (2013)	65 (33 men; 30.2 years)	General population	Resting-state fMRI (RSFC)	EQ-i (self-report mixed model)	EQ-i scores were negatively related to RSFC between the right vmPFC and right amygdala. For MSCEIT no significant results were observed.
				MSCEIT (performance-based ability model)	
Killgore et al. (2017)	54 (26 men; 30.1 years)	General population	Resting-state fMRI (RSFC)	EQ-i (self-repot mixed model)	The EQ-i scores were not related to brain connectivity.
				MSCEIT (performance-based ability model)	The MSCEIT total scores were negatively related to within-network connectivity in the basal ganglia/limbic network and the posterior DMN.
					The MSCEIT total scores were negatively related to between-network connectivity in the relationship between the anterior DMN and the basal ganglia/limbic network and between the posterior DMN and the basal ganglia/limbic network.
Kong et al. (2015)	294 (136 men; 21.56 years)	University students	Resting-state fMRI (fALFF)	WLEIS (self-report ability model)	EI levels were positively related to fALFF in the right amygdala, right posterior superior temporal gyrus, and right thalamus.
Pan et al. (2014)	161 (70 men; 19.40 years)	University students	Resting-state fMRI (ALFF)	WLEIS (self-report ability model)	EI total scores were negatively related to ALFF in the right cerebellum and right fusiform gyrus and positively in the left posterior CC, bilateral SMA and pre-SMA, and right precuneus.
Takeuchi et al. (2013b)	248 (126 men; 21.1 years)	University students	Resting-state fMRI (RSFC)	The Japanese version of the EI scale (self-report mixed model)	EI total scores were positively related to RSFC between the medial PFC and the precuneus and the posterior CC, and between the left anterior insula and the dlPFC.
					The intrapersonal dimension was negatively related to RSFC between the medial PFC and the right dlPFC.
					The interpersonal dimension was positively related to RSFC between the medial PFC and the calcarine cortex.
Xue et al. (2024)	267 (111 men; 22.4 years)	University students	Resting-state fMRI (fALFF)	EQ-i (self-report mixed model)	EI levels were positively related to fALFF in the insula, parahippocampal gyrus, superior temporal gyrus, and superior parietal lobule.
Zanella, Monachesi & Grecucci (2022)	79 (56 men; age range: 20–35 years)	General population	Resting-state fMRI (BOLD temporal variability)	TEIQue-SF (self-report mixed model)	Total EI scores were positively related to BOLD temporal variability within a sensorimotor network (SMA, supramarginal gyrus, inferior frontal gyrus, and insular cortex).
Zhang et al. (2023)	231 (111 men; 18.5 years)	High school students	Resting-state fMRI (fALFF)	WLEIS (self-report ability model)	Levels of EI were negatively related to fALFF in the bilateral medial OFC.
Knyazev, Mitrofanova & Bocharov (2013)	48 (26 men; age range: 18–30 years)	Not specified	EEG (time-frequency and source analysis)	Barchard’s Emotional Intelligence (self-report mixed model)	Participants with high EI, compared with low EI, presented a stronger theta band synchronization in the right fusiform gyrus for angry faces and in the posterior cingulate gyrus for happy faces during the early facial processing stage.
					At a later stage of processing, participants with high EI showed a stronger theta band synchronization in the left PFC for happy faces and a lower theta band in the anterior cingulate gyrus for angry faces.

**Note:**

VHIS, Vietnam head injury study; GM, grey matter; WM, white matter; VBM, voxel-based morphometry; FA, fractional anisotropy; MD, mean diffusivity; RSFC, resting-state functional connectivity; ALFF, amplitude of low-frequency fluctuations; fALFF, fractional amplitude of low-frequency fluctuations; CC, cingulate cortex; dlPFC, dorsolateral prefrontal cortex; OFC, orbitofrontal cortex; SMA, supplementary motor area; vmPFC, ventromedial prefrontal cortex; SMH, somatic marker hypothesis; DMN, default mode network.

### Lesion studies

Four studies investigated the neural correlates of EI by examining individuals with brain lesions. Three of these articles were based on samples of individuals with focal brain damage from the Vietnam Head Injury Study (VHIS) register (Barbey, Colom & Grafman, 2014; Krueger et al., 2009; Operskalski et al., 2015). The fourth study explored individuals with lesions in brain areas related to the somatic marker hypothesis (SMH) neural circuitry (Bar-On et al., 2003).

Barbey, Colom & Grafman (2014) examined 152 American male combat veterans with focal brain damage (M_age_ = 58.1) from the VHIS. After controlling for cognitive intelligence and personality traits, the study found that lower EI levels (measured using the MSCEIT, an instrument from the performance-based ability model) were associated with selective lesions in the right orbitofrontal cortex (OFC), the left inferior and superior parietal cortex, and the superior longitudinal/arcuate, superior fronto-occipital, and uncinate fasciculi. Moreover, the results revealed that EI shared anatomical substrates with cognitive intelligence and personality traits in the perisylvian language network, dorsolateral prefrontal cortex (dlPFC), temporo-parietal junction, and posterior temporal cortex. The authors suggest that the neural bases of EI might involve a broader network of social knowledge implied in the integration of cognitive, social, and affective processes.

Operskalski et al. (2015), using the VHIS registry, explored the neural correlates of the different branches of EI. The sample consisted of 130 brain-damaged male veterans (M_age_ = 59), and EI was assessed using the MSCEIT (performance-based ability model). The study found that lesions in a set of GM and WM brain regions were associated with impairments in the EI branches of perceiving and managing emotions. Both branches shared a neural network characterized by the left temporal pole, left superior temporal gyrus, left insular cortex, and left superior longitudinal fasciculus (SLF). However, specific regions were implicated in each branch: lesions in the left medial ventral temporal cortex, left fusiform gyrus, right middle frontal gyrus, and left uncinate fasciculus were linked to perceiving emotions, while damage to the right OFC, right temporal pole, left posterior temporal lobe, left posterior hippocampus, left parietal cortex, and left inferior longitudinal fasciculus (ILF) were related to impairments in managing emotions. The authors concluded that while the perceiving and managing EI branches rely on distinct neural mechanisms, they depend on a common core of brain structures involved in social cognition and emotional processing.

Krueger et al. (2009) also worked with a sample of male veterans from the VHIS, comparing 17 individuals with dorsolateral prefrontal cortex (dlPFC) damage (M_age_ = 57.3) and 21 with ventromedial prefrontal cortex (vmPFC) damage (M_age_ = 57.9) against a control group of 29 individuals without a head injury (M_age_ = 58.3). EI levels were assessed using the MSCEIT (performance-based ability model) and groups were matched for age, education, and cognitive intelligence. The study found that the vmPFC and dlPFC are integral to the neural system underlying EI, with damage to these areas impairing EI abilities independently of cognitive intelligence. Interestingly, the authors observed a double dissociation, where performance in different EI competencies relied on separate neural prefrontal cortex substrates. Specifically, while vmPFC damage impaired the strategic aspects of EI (*i.e*., the understanding and management of emotions), dlPFC damage affected the experiential aspects (*i.e*., the perception of emotional information and its integration into thinking).

Bar-On et al. (2003) compared the EI levels of 12 patients (7 men, M_age_ = 43.5) with damage in brain areas associated with the SMH circuitry (vmPFC, amygdala, and insula) with those of 11 patients (4 men; M_age_ = 47.1) with lesions in brain structures outside this neural circuitry. The two groups were matched for age, gender, and education. EI was measured using the EQ-i (a self-report mixed model questionnaire), and all patients were also assessed for cognitive intelligence and decision-making performance. The results showed that the group of patients with lesions in the SMH circuitry showed lower levels of EI and impaired decision-making. However, no group differences were found in cognitive intelligence.

Synthesizing the literature from lesion-based studies, the findings emphasize the critical role of brain regions in the social cognition network (SCN) and the SMH circuitry. These areas are implicated in the coordination of cognitive, emotional, and social processes, with key GM regions such as the OFC, dlPFC, vmPFC, amygdala, and insula, as well as WM tracts such as the superior longitudinal fasciculus, being particularly important. Interestingly, the findings also suggest that while the neural bases of EI share some anatomical overlap with those of cognitive intelligence, the two constructs also rely on specific mechanisms that can be dissociated. This is evidenced by cases where damage to brain areas impairs EI but does not necessarily affect cognitive intelligence. Additionally, several studies indicate that specific brain areas are associated with impairments in particular branches of EI, suggesting that, while there is a shared core network, different EI competencies may involve distinct neural substrates.

### Structural MRI: grey matter

Seven structural MRI studies focused on grey matter (GM). Six used voxel-based morphometry (VBM) to assess GM volume or density, and the other explored neural network efficiency through the morphological similarity of the cortical surface.

Koven et al. (2011) employed VBM to explore differences in GM volume associated with the three specific components of EI assessed by the TMMS (an instrument from the self-report ability model). The study involved 30 participants (14 men; M_age_ = 30.0). Age, gender, and education were included as covariates. Lower scores on the “attention to emotions” subscale were associated with reduced GM volume in the superior, middle, ventromedial, and inferior frontal gyri (mostly right-lateralized), bilateral OFC, right frontopolar gyrus, right precentral gyrus, bilateral dorsal anterior cingulate cortex (CC), and right cuneus. The authors argue that these results are consistent with the idea that attention to emotional states engages mechanisms of internally and externally directed attention, self-reflection, and awareness of internal experiences. Lower scores on the “emotional repair” subscale were related to reduced GM volume in the left ACC and right supramarginal gyrus, potentially reflecting poorer inhibitory control and less effective emotion regulation. The “emotional clarity” subscale did not show significant relationships in the VBM analysis.

Takeuchi et al. (2011) used VBM to examine the regional GM density correlates of the three EI factors included in the Japanese version of the EI scale (Fukunishi et al., 2001; self-report mixed model). Their sample comprised 55 participants (42 men; M_age_ = 21.7). Gender, age, and cognitive intelligence were controlled. The findings indicated that the “intrapersonal” factor negatively correlated with GM density in the right anterior insula, right cerebellum, cuneus, precuneus, and a cluster extending from the left medial prefrontal cortex to the left lateral frontopolar cortex. The “interpersonal” factor positively correlated with GM density in the right superior temporal sulcus. The “situation management” factor negatively correlated with GM density in the bilateral vmPFC and positively with the caudal part of the right posterior parietal cortex and the right middle occipital lobe. Each EI factor was associated with distinct brain regions, many of which are involved in the SCN, self-related recognition processes, or the SMH circuitry. The authors suggested that these negative correlations could be explained by the neural efficiency hypothesis or due to patterns of neural development.

Killgore et al. (2012) investigated GM volume differences by VBM in the insula, amygdala, and vmPFC (SMH circuitry) as a function of EI scores assessed by both the self-reported mixed model (EQ-i) and the performance-based ability model (MSCEIT). The sample comprised 36 participants (20 men; M_age_ = 30.0). Gender and age were introduced as covariates in the analysis. The results indicated a positive correlation between EI and vmPFC volume when EI was assessed by the MSCEIT (associated with the strategic area) and the EQ-i (linked to the stress management subscale). Moreover, MSCEIT total scores and its strategic area were positively correlated with insula volume. It is worth noting that the directionality of these correlations was opposite to those reported by Takeuchi et al. (2011).

Tan et al. (2014) analyzed the relationship between EI levels, using the SEIS (self-report ability model), and GM volume (*via* VBM) in a large sample of 328 university students (155 men; M_age_ = 20.0). Gender and age were controlled. The results revealed distinct neural correlates for each dimension of EI. The “monitor of emotions” dimension was positively correlated with the left insula, right OFC, and left superior parietal lobe and negatively correlated with the left cuneus. The “utilization of emotions” dimension was positively correlated with the right parahippocampal gyrus and negatively correlated with the right fusiform gyrus, left middle temporal gyrus, and left superior temporal gyrus. Finally, the “social ability” dimension was positively correlated with the cerebellum, specifically the vermis. The authors linked these findings to the SMH circuitry, cognitive and affective coordination, and processes involved in social perception and semantic association. Negative correlations were considered as indicators of greater neural efficiency.

Xiang et al. (2017) investigated the mediating role of EI (measured with the WLEIS, a self-report ability instrument) in the relationship between GM volumes (measured by VBM) and dispositional envy. The study used a sample of 73 university students (32 men; M_age_ = 21.5). Gender and age were controlled. The results revealed a negative correlation between the dlPFC and EI levels. These findings were replicated with a second sample of 27 participants (11 men; M_age_ = 20.6 years). It should be noted that the VBM analysis for EI focused only on the superior temporal gyrus and dlPFC, the brain areas previously linked to dispositional envy in this study.

He et al. (2018) examined the neural basis of EI (regional GM volumes by VBM) and its relationship with creativity in a university sample of 213 participants (103 men; M_age_ = 20.0 years). EI was assessed using the SEIS (self-report ability model), and gender, age, and cognitive intelligence were controlled. The results revealed a positive relationship between EI total score and GM volume in the right OFC, suggesting that these volumetric differences in the OFC are associated with top-down processes of emotional regulation.

Li et al. (2021) constructed a morphological brain network using several MRI cortical surface parameters, including cortical volume, surface area, and thickness. They observed that the relationship between the efficiency of this network (morphological similarity) and EI was age-dependent. The study involved 65 participants (32 men; mean age = 37.3). EI was assessed using the SEIS (self-report ability model), and gender and education level were controlled in the analyses. A positive correlation between the global efficiency of the network and EI was observed for middle-aged participants, while this correlation was negative for young adults. Additionally, middle-aged participants showed a positive correlation between nodal efficiency and EI the sensory-motor area, temporal pole, inferior parietal sulcus, and, most notably, in regions associated with the default mode network (DMN). Their findings suggest distinct neurodevelopmental trajectories for EI and cognitive intelligence.

A collective analysis of the findings from these studies reveals that, consistent with lesion studies, the OFC, dlPFC, vmPFC, and insula are among the key neural correlates of EI. These areas have been linked to the neural network for social cognition, cognitive and affective coordination, and the SMH circuitry. Moreover, GM differences in the ACC, cerebellum, and some specific regions of the parietal, temporal, and occipital lobe were also observed in several studies. It is important to note that some highlighted areas, such as the dlPFC, vmPFC, insula, and cerebellum, showed either negative or positive correlations with EI, depending on the research. Studies associating reduced GM volumes with higher EI levels considered these results as an indicator of greater neural efficiency, however, the reasons for these variations across studies remain unclear. Finally, specific brain regions were related to distinct dimensions of EI, and, according to Li et al. (2021), the neural correlates of EI could vary with age.

### Structural MRI: white matter

The systematic review identified three articles exploring white matter (WM) correlates of EI. Two studies used fractional anisotropy (FA) to assess the integrity of WM tracts, while the third employed mean diffusivity (MD) to analyze both WM and GM.

Pisner et al. (2017) examined the relationship between WM integrity and EI using FA measurements. The study involved 32 participants (16 men; M_age_ = 29.2), with EI assessed using the MSCEIT (performance-based ability model). Cognitive intelligence, gender, and age were controlled in the analysis. The “understanding emotions” branch of the MSCEIT positively correlated with FA in the left superior longitudinal fasciculus and dorsal corticospinal tract, a finding interpreted by the authors as related to improved sensory and semantic processing of somatic and visual/auditory signals. The “managing emotions” branch correlated positively with FA in the *corpus* callosum (anterior and posterior forceps), cingulum bundle, and uncinate fasciculus. Greater tract integrity was associated with better inter-hemispheric integration of social/emotional information, attentional control mechanisms, and decision-making processes, including somatic markers and top-down emotion regulation.

Takeuchi et al. (2013b) investigated the association between EI levels, as assessed by the Japanese version of the EI scale (self-report mixed model), and differences in FA in a sample of 118 university students (74 men; M_age_ = 21.6). Gender, age, and cognitive intelligence were controlled. Their study found that the “intrapersonal” factor of EI correlated positively with FA in the right anterior insula, while the “interpersonal” factor correlated positively with FA in parts of the inferior longitudinal fasciculus, extending from the right middle occipital lobe to the fusiform gyrus and parahippocampus. The authors proposed that greater WM integrity in the anterior insula could be related to monitoring visceral states and integrating multimodal information (as a part of the SMH circuitry), while the inferior longitudinal fasciculus would be involved in facial perception and emotional information processing.

Takeuchi et al. (2019) assessed 1,207 university students (693 men; M_age_ = 20.8) in EI using the Japanese version of the EI scale (self-report mixed model) and studied both WM and GM integrity of these participants using MD measurements. Gender, age, and cognitive intelligence were controlled. The results revealed that the “intrapersonal” factor of EI correlated negatively with MD in clusters the right putamen, globus pallidum, posterior insula, and fusiform gyrus, and positively with MD in the medial PFC, ACC, and left inferior frontal gyrus. The “interpersonal” factor correlated positively with MD in the precuneus, while the “management” factor correlated positively with MD in the ACC, insula, and lateral prefrontal cortex. These findings highlighted the importance of the dopaminergic system (particularly for the intrapersonal factor and aspects related to self-motivation) and the role of the SMH and SCN.

The reviewed articles demonstrate a wide range of WM tracts and brain regions associated with EI. However, they consistently emphasize the significance of structures implicated in integrating and processing emotional and social information, particularly those linked to the SMH circuitry and the SCN. Moreover, Takeuchi et al. (2019) introduced an additional network associated with the dopaminergic system, which could be related to motivational processes—a key component in the EI model that conceptualizes EI as a trait. Finally, as in previous sections, these studies suggest that different brain regions are implicated depending on the specific dimensions of EI.

### Task-based fMRI

The review identified 10 studies analyzing the neural correlates of EI through task-based fMRI (one study also included a VBM analysis (Karle et al., 2018)). Six studies were based on face processing, two on social reasoning, and one used an interoceptive task. Interestingly, another involved training in EI.

Killgore & Yurgelun-Todd (2007) examined the relationship between EI and brain activation during a fearful-face perception task in 16 children and adolescents (nine men; M_age_ = 11.6). EI was assessed using the EQ-i-Youth version (self-reported mixed model), with gender controlled in the analysis. Higher levels of EI were associated with reduced activation in the vmPFC, amygdala, insula, middle cingulate gyrus, hippocampus, and parahippocampal gyrus in response to fearful faces. Thus, participants with more well-developed EI abilities showed lower reactivity in these regions to fearful stimuli. In addition, higher levels of EI were linked to greater activation in the left cerebellum and right occipital cortex.

Alkozei & Killgore (2015), using a sample of 44 participants (21 men; M_age_ = 30.4), investigated the relationship between levels of EI and neural response to subliminally presented angry faces. EI was measured using the MSCEIT (performance-based ability model). fMRI analysis was restricted to the vmPFC, amygdala, and insula (brain regions involved in emotional response and its regulation) and cognitive intelligence was controlled. The results revealed that higher EI was associated with reduced activation in the left posterior insula while presenting the subliminal angry faces, suggesting that individuals with higher EI abilities show lower emotional reactivity to social threat cues.

Killgore et al. (2013) studied the relationship between EI and brain responses to changes in the perceived trustworthiness of facial expressions in 39 participants (22 men; M_age_ = 29.9). EI was assessed by two instruments: the MSCEIT (performance-based ability model) and the EQ-i (self-reported mixed model). The analyses were limited to brain regions involved in the SMH circuitry. Higher MSCEIT total scores were associated with greater activation in the vmPFC and rostral anterior CC during those conditions in which the facial expressions decreased in trustworthiness. EQ-I scores were not associated with any changes in brain activation.

Quarto et al. (2016) examined the relationship between levels of activation of the SMH circuitry during a social judgment task and EI. Participants were asked to decide whether they would avoid or approach a set of emotional facial expressions. The sample comprised 63 participants (29 men; M_age_ = 29.4). EI was measured using the MSCEIT (performance-based ability model), and gender, age, and cognitive intelligence were controlled. EI scores were related to changes in the activation of the left insula during the social judgment task, but this depended on the type of facial expression. Participants with higher EI showed increased activation in the left insula for fearful faces but decreased activation for angry faces.

Kreifelts et al. (2010) investigated the relationship between EI and the functional neural correlates of audiovisual integration of emotional signals from voices and faces. The sample consisted of 24 participants (12 men; M_age_ = 26.6), and EI was assessed by the SEIS (self-report ability model). Activation in the right posterior superior temporal sulcus was positively correlated with EI scores during audiovisual integration, which the authors linked to social cognitive functions and better processing of social information.

Karle et al. (2018) investigated whether differences in EI levels are reflected in the neural processing of human voices and faces. A sample of 85 participants (42 men; M_age_ = 25.4) was assessed for EI using the MSCEIT (performance-based ability model). Gender and age were introduced as covariates in the analyses. The findings revealed a positive correlation between EI scores and cerebral voice sensitivity (the differential neural response to voices as compared to other non-human vocal sounds) in the left amygdala, left anterior insula, and left inferior frontal gyrus. Moreover, high EI was linked to enhanced functional connectivity between the left insula and the middle part of the right temporal voice area. Concerning cerebral face sensitivity (the neural response to human faces compared to other visual stimuli), a negative relationship between EI scores and the right fusiform face area was observed. In addition to the fMRI analyses, this study also explored GM volumes (using VBM), revealing that MSCEIT scores were positively correlated with the volume of the right OFC and left insula and negatively correlated with the right fusiform face area. The authors suggested that individuals with higher EI show increased sensitivity to human voices, while the opposite pattern is observed for the processing of faces.

Dobrushina et al. (2020) explored the functional brain basis of the relationship between EI and interoceptive accuracy using a heartbeat detection task. The sample consisted of 30 women (M_age_ = 51), who were assessed for EI using the MSCEIT (performance-based ability model). Age was controlled in the analysis. The results revealed that activation of the right anterior insula during the interoceptive task positively correlated with EI levels and heartbeat detection accuracy. The authors propose that the right anterior insula plays a mediating role between interoception and EI.

Reis et al. (2007) studied neural activation during a social exchange reasoning task (using the Wason Card Selection Task) as a function of individual differences in EI assessed by the MSCEIT (performance-based ability model). The sample comprised 16 participants (6 men; M_age_ = 21.7). Higher EI was associated with decreased neural activity during social reasoning in the left anterior temporal and left frontopolar regions. The authors suggested that individuals with lower EI experienced greater difficulty in reasoning about the social situations presented, necessitating compensatory recruitment of these brain areas.

Deak et al. (2022) explored the role of EI in emotional perception and regulation processes and the functional neural mechanisms underlying this relationship. A sample of forty young adults (19 men; M_age_ = 21.0) performed a cognitive emotion regulation task inside an MRI scanner where they were asked to reappraise the emotional content of social scenes. EI levels were assessed using the SEIS (self-report ability model). Participants with higher EI reported more unpleasant emotions when visualizing negative stimuli but found it easier to reappraise these scenes. At the neural level, these participants exhibited higher activation in the hippocampus, parahippocampal gyrus, posterior CC, insula, and superior temporal lobe during the negative scenes, suggesting a stronger affective experience. Regarding the reappraisal process, EI levels were negatively related to changes in activation in the medial frontal gyrus, temporal areas, and CC and positively associated with vision-related regions and the superior parietal lobe. These latter findings suggest a better allocation of cognitive resources for effective emotional regulation.

Hansenne et al. (2014) conducted an experimental study examining changes in brain functionality following EI training. Brain activation was measured during an emotional regulation task where participants were asked to increase, decrease, or not to modulate their emotional reactions to various images. Thirty-six women (M_age_ = 21) were divided into two groups (18 participants each): a control group (drama improvisation training) and an experimental group trained in emotional competencies. After training, the experimental group, compared with the control group, scored significantly higher in EI (assessed by the TEIQue-Short form; self-report mixed model) and demonstrated better modulation of their arousal during the emotional task. Neuroimaging results revealed that the experimental group showed lower activation than the control group in the bilateral inferior parietal lobe, right precentral gyrus, and intraparietal sulcus when decreasing negative emotions and in the right middle frontal gyrus, left OFC, and right frontopolar cortex when increasing positive emotions. These lower activation levels in the EI training group were interpreted as evidence of greater neural efficiency, reflected in improved emotional regulation with less cognitive effort.

Analyzing the reviewed task-based fMRI studies reveals a particular emphasis on the relationship between EI and the insula. For example, Killgore & Yurgelun-Todd (2007) and Alkozei & Killgore (2015) demonstrated that individuals with higher EI showed a decreased neural response in the insula when viewing negative emotional faces. Quarto et al. (2016) further reported that higher EI was associated with lower neural activation in response to angry faces and higher activation for fearful faces. Studies that did not involve face processing also found a positive association between activation in the insula and EI levels (Deak et al., 2022; Dobrushina et al., 2020; Karle et al., 2018). Therefore, while the involvement of the insula in EI is clear, the direction of its activation appears to depend on the specific task. As in previous sections, other brain regions commonly implicated in emotional processing and SMH circuitry, such as the vmPFC, amygdala, or CC, were also related to EI in several studies. In addition, areas such as the hippocampus, parahippocampal gyrus, and certain temporal areas have been highlighted. Finally, it is worth noting that the experimental study involving EI training (Hansenne et al., 2014) found that improved emotional regulation performance was associated with reduced activation in prefrontal and parietal regions, a pattern of results that is consistent with the neural efficiency hypothesis.

### Resting-state fMRI

We identified nine studies that investigated the neural basis of EI using resting-state fMRI. Of these, five employed resting-state functional connectivity (RSFC) as the primary method of analysis, one used amplitude of low-frequency fluctuations (ALFF), and three used fractional amplitude of low-frequency fluctuations (fALFF).

Takeuchi et al. (2013a), following their previous works, explored the neural structures involved in the SCN and SMH circuitry to explain the brain mechanisms underlying EI. Specifically, they investigated RSFC between the mPFC and bilateral anterior insula—two key nodes related to these networks—and other regions across the brain. Their study comprised 248 participants (126 men; M_age_ = 21.1), with EI assessed using the Japanese version of the EI scale (self-report mixed model). Cognitive intelligence, gender, and age were controlled. The results revealed that total EI scores were positively related to functional connectivity between the medial PFC and the precuneus and posterior CC and between the left anterior insula and the middle part of the right dlPFC. When looking at specific EIS dimensions, the intrapersonal dimension was negatively related to functional connectivity between the medial PFC and the anterior part of the right dlPFC, while the interpersonal dimension was positively related to RSFC between the medial PFC and the calcarine cortex.

Killgore (2013) studied the effects of sleep curtailment on prefrontal-amygdala RSFC and its relationship with EI levels. A sample of 65 participants (33 men; M_age_ = 30.2) was assessed for EI by using two different approaches: the self-report mixed model (EQ-i) and the performance-based ability model (MSCEIT). The results revealed that longer self-reported sleep duration and higher EQ-i scores (but not MSCEIT scores) were associated with stronger negative functional connectivity between the right ventromedial prefrontal cortex (vmPFC) and the right amygdala. The authors concluded that better sleep facilitates top-down regulatory capacity, a critical component of EI.

Killgore et al. (2017) examined the relationship between EI and RSFC patterns within and among four intrinsic brain networks previously shown to be involved in emotional processing. Fifty-four participants (26 men; M_age_ = 30.1) were assessed for EI using the EQ-i (self-repot mixed model) and MSCEIT (performance-based ability model). The results revealed that MSCEIT total scores were negatively related to within-network functional connectivity in a basal ganglia/limbic network and the posterior DMN. Moreover, regarding the functional connection among networks, MSCEIT total scores were negatively related to the correlation between the anterior/posterior DMN and the basal ganglia/limbic networks. In contrast, no significant relationships were found between EQ-I scores and brain connectivity within or among the four networks. The authors propose that EI, when understood as an ability, is linked to changes in the relationships between brain regions involved in emotional responses and those implicated in self-reflective cognition and emotional regulation.

Zanella, Monachesi & Grecucci (2022) explored the relationship between BOLD temporal variability (as measured by RSFC), and individual differences in both EI and emotion regulation strategies. Seventy-nine participants (56 men; age range: 20–35 years old) were assessed for EI using the TEIQue-Short form (self-report mixed model). The findings revealed that participants with higher EI showed an increased BOLD temporal variability within the sensorimotor network, which included regions such as the supplementary motor area (SMA), supramarginal gyrus, inferior frontal gyrus, and insular cortex.

Bajaj & Killgore (2021) explored the relationship between EI and effective brain connectivity within four networks: the DMN, dorsal attention network, control-execution network, and salience network. A sample of 55 participants (26 men; M_age_ = 30.1) was assessed using the MSCEIT (performance-based ability model). Gender, age, and education were controlled in the analysis. The results revealed no significant association between effective connectivity strength and total EI in any of the four networks. However, when examining specific MSCEIT branches, the abilities to facilitate and manage emotions were significantly associated with effective connectivity in the control-execution network. Specifically, negative and positive correlations were found between the connection of the right and left anterior prefrontal cortex, and a negative correlation was found between the superior parietal cortex and the right anterior prefrontal cortex. The “understanding emotions” branch was related to effective connectivity strength in the salience network, with a negative association for the connection between the right insula and the right anterior prefrontal cortex and a positive association for the connection between the left insula and the dorsal anterior CC.

Pan et al. (2014) investigated whether variations in EI are linked to differences in resting state fMRI using the ALFF index. A sample of 161 university students (70 men; M_age_ = 19.40), was assessed using the WLEIS (self-report ability model), and gender and age were introduced as covariates in the analysis. The results revealed negative correlations between EI total scores and ALFF in the right cerebellum and right fusiform gyrus and positive correlations in the left posterior CC, bilateral SMA and pre-SMA, and right precuneus. The authors linked these findings with two neural networks implied in social-emotional processing and top-down control, which are responsible for the correct perception, understanding, and control of emotions.

Zhang et al. (2023), using the fALFF approach, investigated the neural basis of EI and its relationship with anxious and depressive symptoms. A sample of 231 high school students (111 men; M_age_ = 18.5) were assessed for EI using the WLEIS (self-report ability model), with gender and age controlled. The results showed that EI levels were negatively related to fALFF values in the bilateral medial OFC.

Xue et al. (2024) explored how EI influences the relationship between brain fALFF values and social interaction anxiety. A sample of 267 university students (111 men; M_age_ = 22.4) was assessed for EI using the EQ-i (self-report mixed model). Gender and age introduced as confounding covariates. The study revealed a positive correlation between EI and fALFF values in the insula, parahippocampal gyrus, superior temporal gyrus, and superior parietal lobule. It is important to note that this analysis was restricted to those regions where fALFF was related to social interaction anxiety.

Kong et al. (2015) examined the mediating role of EI in the relationship between spontaneous neural activity at rest, measured by fALFF, and subjective well-being. The study comprised 294 university students (136 men; M_age_ = 21.56), with EI assessed using the WLEIS (self-report ability model). Gender and age were controlled in the analysis. The results indicated that EI was positively correlated with fALFF values in the right amygdala, right posterior superior temporal gyrus, and right thalamus. As in the previous study, the brain regions were restricted to those where fALFF values were associated with well-being.

To summarize, two studies based on RSFC (Killgore et al., 2013, 2017) highlighted the importance of several brain regions involved in emotional response processing and top-down emotional regulation as part of the neural correlates of EI, emphasizing changes in functional connectivity between the vmPFC and the amygdala, as well as the involvement of brain networks such as the DMN and the basal ganglia/limbic network. Interestingly, one of these studies (Killgore et al., 2013) reported significant neural correlates using the EQ-I (an EI mixed model instrument) but not with the MSCEIT (an EI ability model instrument), whereas the other study (Killgore et al., 2017) found the opposite pattern. In addition, Takeuchi et al. (2013a) reinforced the importance of regions associated with the SCN and the SMH circuitry in explaining EI, while Zanella, Monachesi & Grecucci (2022) and Bajaj & Killgore (2021) pointed to the involvement of the sensorimotor network, control-execution network, and salience network. The other four articles focused on spontaneous neural activity during the resting state using amplitude low-frequency fluctuation methods (ALFF and fALFF). These studies supported the involvement of brain regions associated with social-emotional processing and regulation, as noted in earlier sections of this review. These regions included the amygdala, insula, OFC, CC, superior temporal gyrus, superior parietal lobule, thalamus, cerebellum, fusiform gyrus, SMA, and precuneus.

### EEG

Only one EEG study met the inclusion criteria for this review, as the others failed to include brain source localization analysis. Knyazev, Mitrofanova & Bocharov (2013) investigated differences in cortical oscillations when evaluating emotional faces (hostility-friendliness) as a function of EI levels. The study included 48 participants (26 men; age range: 18–30), and EI was assessed using Barchard’s Emotional Intelligence scale (Barchard, 2001; self-report mixed model). Age was controlled as a covariate. In an early processing stage (100–500 ms after face stimulus onset), participants with higher EI, compared with those with lower EI, showed stronger theta band synchronization, primarily localized in the right fusiform gyrus for angry faces and in the posterior cingulate gyrus for happy faces. In the later stage (from 500 to 870 ms), higher EI was associated with increased theta synchronization in the left PFC for happy faces. In contrast, for angry faces, higher EI was related to reduced theta synchronization in the anterior cingulate gyrus. Based on these findings, the authors suggested that individuals with high EI are more sensitive to emotional content in the early stages of perception, but possess better emotional regulation abilities, enabling them to modulate their emotions by selectively increasing or decreasing their emotional responses.

### Assessment of study quality

Given the diversity of study designs included in this systematic review, ranging from correlational and comparative non-experimental studies to experimental studies, we prefer to provide a narrative summary rather than applying standardized quality assessment tools such as the Newcastle–Ottawa Scale or CONSORT. This approach allows us to conduct a comprehensive evaluation while addressing the difficulties that arise when applying standardized tools to compare quality aspects across heterogeneous study designs. Our narrative assessment focuses on the main methodological aspects related to study quality, including adequate sample selection, validity of the measures used, control of confounders, and research design rigor.

The studies included in this systematic review employed adequately sized samples, with the exception of two that comprised only 16 participants. However, a substantial proportion of them focused exclusively on university students, specifically 11 studies. Additionally, 27 out of 34 studies involved participants aged 30 years or younger. These characteristics limit the generalizability of the findings to broader community samples. Regarding gender distribution, two studies consisted solely of women, and three solely of men, while the remaining samples were generally well-balanced. Across all studies, the total number of participants was 4,434, comprising 2,422 men and 2,012 women.

The majority of the measures used to assess EI corresponded to the most widely recognized instruments within each of the EI models (the MSCEIT in the performance-based ability model; the SEIS, TMMS, and WLEIS in the self-report ability model; and the EQ-i and TEIQue in the self-report mixed model; see Bru-Luna et al., 2021). These instruments have demonstrated good psychometric properties and, overall, have received support in the literature. However, we identified the use of two instruments with more limited support: Barchard’s Emotional Intelligence Test, which was employed in one study, and the Japanese version of the EI Scale, which was used in four studies.

Finally, with respect to the research designs and statistical analysis procedures, the limited use of experimental methodology is particularly notable. Only one study employed an experimental design, in which participants received EI training and were compared with a control group. The remaining studies were primarily correlational, except for two that adopted a non-experimental comparative design. Moreover, while some studies employed stringent corrections for multiple comparisons, others applied too liberal adjustments. Another point worth noting is the consideration of confounding factors: cognitive intelligence was included as a covariate in 10 studies, level of education in three, and personality traits in one. Given the demonstrated overlap of EI with personality factors—particularly within the mixed model—and cognitive intelligence (Joseph & Newman, 2010; Van der Linden et al., 2017), it would have been advisable to include these constructs in a greater number of studies.

## General discussion

This systematic review aimed to identify and synthesize the existing literature on the neural bases of EI. After a comprehensive search and study selection process, we identified 34 peer-reviewed articles that used various neuroimaging techniques to explore the neural correlates underlying EI. These included four lesion-based studies, 10 structural MRI studies (seven focused on GM and three on WM), 10 task-based fMRI studies, nine resting-state fMRI studies, and one study using EEG-based brain source localization.

Regardless of the neuroimaging technique employed, the studies consistently identified a set of brain regions involved in the integration of emotional information and cognition, particularly in processes related to social cognition. Many of these studies point to the relevance of the SMH neural circuitry and the SCN in explaining EI. Key brain regions common to both networks include the vmPFC, OFC, DLPFC, CC, and insula (Alcalá-López et al., 2018; Bechara, 2011; Bechara & Damasio, 2005; Krendl & Betzel, 2022; Pelphrey & Carter, 2008). In the next sections, we will discuss the most salient findings from this systematic review.

### Main brain correlates of EI

The literature reviewed indicates that the neural correlates of EI are primarily associated with the following brain regions: the insula (17 articles), the CC (10 articles), the superior temporal sulcus/gyrus (eight articles), the OFC (eight articles), the vmPFC (eight articles), the fusiform gyrus (six articles), the cuneus and precuneus (six articles), the amygdala (five articles), and the dlPFC (four articles). Below, we discuss these regions in more detail.

Integrating the primary findings of lesion studies shows that damage to the vmPFC, OFC, dlPFC, and insula leads to significant impairments in EI (Barbey, Colom & Grafman, 2014; Bar-On et al., 2003; Krueger et al., 2009; Operskalski et al., 2015). Concerning GM structure, greater GM volume in the OFC and the insula was generally associated with higher levels of EI (He et al., 2018; Killgore et al., 2012; Koven et al., 2011; Tan et al., 2014), while the cuneus and vmPFC volumes exhibited positive and negative relationships with EI, depending on the study (Koven et al., 2011; Killgore et al., 2012; Takeuchi et al., 2011; Tan et al., 2014). Task-based fMRI studies also identified the vmPFC, OFC, dlPFC, amygdala, insula, and CC as critical regions, but the directionality of these relationships often depended on the type of task and stimuli presented. For instance, studies by Deak et al. (2022), Dobrushina et al. (2020), and Karle et al. (2018) reported greater activation in the insula in emotionally intelligent participants. In contrast, Alkozei & Killgore (2015) and Killgore & Yurgelun-Todd (2007) found the opposite, with less activation of the insula associated with higher EI levels. Studies associating higher EI with lower levels of brain activation, as well as those showing reduced GM volume, often rely on the neural efficiency hypothesis to explain these findings. This hypothesis posits that individuals with greater EI abilities require fewer neural resources due to more efficient brain functioning. The reduced brain activation was also typically attributed to lower emotional over-reactivity to certain stimuli, resulting from improved top-down emotional regulation (Alkozei & Killgore, 2015; Hansenne et al., 2014; Killgore & Yurgelun-Todd, 2007; Megías-Robles et al., 2019). Thus, while future fMRI studies are needed to confirm these hypotheses, differences in directionality could depend on task demands and reactivity to various emotional stimuli. In resting-state fMRI studies, higher levels of EI were associated with negative functional connectivity between the vmPFC and the amygdala (Killgore, 2013), and positive functional connectivity between the insula and the dlPFC, as well as between the mPFC and the precuneus and posterior CC (Takeuchi et al., 2013a). Moreover, changes in fALFF as a function of EI levels were revealed in the OFC (Zhang et al., 2023), insula (Xue et al., 2024), and amygdala (Kong et al., 2015). Finally, one EEG study also found evidence for the involvement of the fusiform and cingulate gyri in EI, particularly in emotional perception and regulation (Knyazev, Mitrofanova & Bocharov, 2013).

Regarding the WM tracts, the findings are more limited, but the reviewed literature underscores the importance of the SLF. Both lesions (Barbey, Colom & Grafman, 2014; Operskalski et al., 2015) and reduced WM integrity (Pisner et al., 2017) in this fasciculus were associated with lower EI levels. Pisner et al. (2017) linked these deficits to impairments in sensory/semantic processing, while Barbey, Colom & Grafman (2014) associated them with social information processing. In any case, given the extensive and distributed brain networks that involve the SLF, future studies should explore in more detail the role of this fasciculus in EI. Additionally, several studies have also related EI to the integrity of other WM tracts, including the ILF (Operskalski et al., 2015; Takeuchi et al., 2013b) and the uncinate fasciculus (Barbey, Colom & Grafman, 2014; Operskalski et al., 2015; Pisner et al., 2017).

Many of the reviewed articles highlight the involvement of several of these brain regions in the SMH neural circuitry, emphasizing the role of the somatic marker as a fundamental component of the basis of EI. The SMH posits that decision-making is guided by emotional and bodily states (Bechara, 2011; Bechara & Damasio, 2005). According to this model, the amygdala triggers emotional and bodily states in response to rewards and punishments from the environment. After learning, the vmPFC can elicit these somatic states, which become associated with mental representations of behavior and its consequences. The insula, in turn, integrates, perceives, and monitors these emotional states, guiding future decisions (Bechara & Damasio, 2005; Naqvi, Shiv & Bechara, 2006). The CC and the dlPFC are other two areas involved in the SMH neural circuitry, with the anterior part of the CC related to processing emotional salience and the resolution of emotional conflict (Etkin et al., 2006; Killgore et al., 2013), while the dlPFC is associated with working memory, attention, inhibitory control, and emotion regulation strategies (Bechara & Damasio, 2005; Buhle et al., 2014; Kane & Engle, 2002). Finally, the OFC also frequently appears in the reviewed literature. Its role is emphasized in the processing of rewards and punishments, representing affective values, and integrating and regulating emotional experiences (Tan et al., 2014; Zhang et al., 2023). However, it is important to note that there is some controversy in the SMH literature regarding the use of the terms OFC and vmPFC. These terms have often been used interchangeably, as the vmPFC sometimes refers to regions that include medial portions of the OFC (Bechara, 2011; Dunn, Dalgleish & Lawrence, 2006).

The other brain network frequently highlighted in the reviewed articles is the SCN (for a detailed description of this network, see Alcalá-López et al. (2018), Krendl & Betzel (2022), or Pelphrey & Carter (2008)). The presence of a neural network dedicated to social processing is expected, given the necessity of integrating emotional information for social interactions and the demonstrated predictive capacity of EI for performance in social environments (Brackett, Rivers & Salovey, 2011; Lopes et al., 2005; Ruiz-Aranda et al., 2012). Several brain regions involved in the SCN, which have also been linked to EI in the reviewed studies, overlap with those identified in the SMH neural circuitry. These regions include the vmPFC, OFC, CC, and insula. Additionally, other areas, such as the cuneus/precuneus, fusiform gyrus, and superior temporal sulcus/gyrus, have also been implicated in both EI and SCN functions. Although more research is needed to fully understand the specific roles of these latter brain areas in EI, the literature mostly agrees on their involvement in mechanisms for monitoring and regulating the affective aspects of social behavior, reasoning about mental states (both one’s own and others), and coordinating cognitive, social, and emotional processes. For example, the precuneus is of particular interest in EI research due to its involvement in consciousness, theory of mind, and self-referential processes (Dadario & Sughrue, 2023; Cavanna & Trimble, 2006). The fusiform gyrus is well-known for its role in facial perception and the processing of facial emotions, both of which are crucial in social interactions (Grill-Spector et al., 2017; Sabatinelli et al., 2011). Similarly, the superior temporal sulcus is suggested to be important for the perception of social and emotional signals and for representing mental states (Pelphrey & Carter, 2008; Takeuchi et al., 2011).

In addition to the SMH and SCN, several studies suggest the contribution of other neural networks, such as the salience network and the DMN. Regarding the salience network, the ACC and the anterior insula have been identified as key regions responsible for attending to and processing emotionally salient information—an essential process for acquiring better emotional abilities (Bajaj & Killgore, 2021; Deak et al., 2022; Dobrushina et al., 2020; Takeuchi et al., 2019). On the other hand, studies examining RSFC and morphological similarity as a function of EI levels in regions such as medial PFC, precuneus, and posterior cingulate cortex have established a connection between these outcomes and the DMN (Killgore et al., 2017; Li et al., 2021; Takeuchi et al., 2013a). In this regard, Smith et al. (2018) proposed a neuro-cognitive model of EI^1^ 1This neuro-cognitive process model of EI is not based on EI measures per se, but rather on literature studying cognitive-emotional processes including the abilities to recognize/understand and generate/regulate emotions. in which both the salience network and DMN play critical roles, being these implicated in automatic emotional attention, the use of bodily state information to direct attention to emotionally salient stimuli, and the conceptualization of emotions. Although these findings are promising, further research is needed to fully understand the specific role of both networks in EI and their convergence with the SMH and SCN.

### Further brain correlates of EI

In addition to the main brain regions discussed above, several other areas were identified in the reviewed articles as potentially relevant to understanding the neural basis of emotional EI, although a clear consensus between studies is still lacking. These areas could offer interesting insights into the brain mechanisms underlying EI.

The cerebellum was highlighted in four studies. While the cerebellum is primarily known for its role in motor coordination, previous research has also demonstrated its involvement in emotional processing, including perception, encoding, and regulation of emotional information (Adamaszek et al., 2017; Fastenrath et al., 2022). In our review, Takeuchi et al. (2011) and Tan et al. (2014) reported changes in GM structure, while Killgore & Yurgelun-Todd (2007) and Pan et al. (2014) found differences in activation in the cerebellum that were related to EI levels. It is important to note that these studies reported varying correlation directions and different regions of the cerebellum implicated in each case.

The parahippocampal gyrus and hippocampus were highlighted in seven of the studies. The authors emphasized their role in identifying social contexts and forming contextual associations, enabling individuals to interpret their own and others’ perceptions and emotional states through the use of autobiographical memory (Deak et al., 2022; Operskalski et al., 2015; Smith et al., 2018).

The inferior frontal gyrus, the frontopolar gyrus, and the superior parietal lobule were each referenced in four articles, though the reasons for their involvement in EI remain inconclusive. Some studies pointed out the role of the inferior frontal gyrus in inhibitory control (Takeuchi et al., 2019), while the frontopolar gyrus may be linked to social reasoning (Takeuchi et al., 2011). The superior parietal lobule was suggested to be involved in sensorimotor integration related to emotional experiences (Tan et al., 2014). However, the literature agrees that further research is needed to confirm their potential roles in emotional processing.

### Comparison of EI models

Having reviewed the main brain structures and networks linked with EI in the literature, it is important to consider that the reviewed studies employed a wide variety of measurement instruments from different EI models (see Joseph & Newman, 2010): 14 articles used the performance-based ability model, 10 used the self-report ability model, and 13 used the self-report mixed model. As detailed in the Introduction, these models differ in their theoretical conceptualization of EI (ability or mixed EI) and in the measurement instruments used (self-report or performance-based), showing weak convergent validity between them. Thus, it is unsurprising that differences could emerge between models at the neural level. However, closer evaluation of the neural findings observed for each model in this systematic review reveals no consistent pattern that allows clear differentiation between them. All the main brain regions that had been associated with EI (see ‘Main brain correlates of EI’) were observed across the three models. The only exceptions were the cuneus and precuneus, which were not identified in the performance-based ability model. The CC also appeared less prominent in this model. In addition, the amygdala was relatively more frequently reported in the mixed self-report model, whereas the insula was less prevalent in the self-report ability model. Nevertheless, drawing firm conclusions from these observations is problematic due to the considerable variability in neuroimaging techniques, methodologies, and the specific brain regions examined across studies for each EI model.

To address this issue, a more effective approach would be to focus on those studies that have assessed participants using multiple EI models. Among the articles reviewed, four studies used both the MSCEIT (performance-based ability model) and the EQ-I (self-report mixed model) (Killgore, 2013; Killgore et al., 2012, 2013, 2017). Examination of these studies also yields inconclusive results. While they consistently identify the vmPFC as a key region in EI, they diverge on the EI model with which this area is associated, with different studies attributing the vmPFC to different models. Given these mixed findings and the well-documented theoretical distinctions between the models (Gutiérrez-Cobo, Cabello & Fernández-Berrocal, 2016; Joseph & Newman, 2010), identifying the neural correlates of EI according to each model remains a promising avenue for further research.

### Association with cognitive intelligence

Another interesting question to emerge from the reviewed articles is whether EI and cognitive intelligence rely on different neural substrates or share common regions. Several studies have revealed that certain brain lesions impairing EI did not compromise cognitive intelligence (Bar-On et al., 2003; Krueger et al., 2009). Furthermore, many of the neural correlates of EI identified in this review were found in studies that controlled for cognitive intelligence in the analyses or study design. These findings suggest that while EI may depend on and share anatomical substrates with cognitive intelligence (Barbey, Colom & Grafman, 2014), the two constructs could be dissociated. In particular, EI seems to rely on specific mechanisms for processing social and emotional information. Nevertheless, further investigation is needed on this topic, as the current evidence remains inconclusive.

### Limitations and future lines of research

It is important to acknowledge limitations in the literature reviewed, which should be addressed in future research. First, most studies employed samples comprising university students. Although this approach allows for recruiting larger samples, it introduces potential bias, as the participants are limited to young people, and there is a demonstrated relationship between higher academic performance and higher EI (Brackett, Rivers & Salovey, 2011; MacCann et al., 2020). Further research should aim to include more representative samples of the general population and consistently take sociodemographic factors such as age and gender into account (Cabello et al., 2016; Megías-Robles et al., 2024). Moreover, emotional intelligence is shaped and expressed differently across cultural contexts, and therefore, cross-cultural variations should be carefully considered (Chen et al., 2025; Park et al., 2025). Second, significant methodological differences exist across studies, regardless of the neuroimaging techniques employed. A considerable number of studies restricted their analysis to specific brain regions, leading to the identification of certain regions only in studies that conducted whole-brain analyses. Furthermore, some studies showed a strong methodological rigor by accounting for potentially confounding variables such as cognitive intelligence and personality traits, while others lacked such controls or employed overly liberal corrections for multiple comparisons. Finally, it is worth noting that even within the same EI model, measurement instruments differ in the dimensions or factors they assess—especially within the mixed model. The use of diverse EI models, varying dimensions of the measurement instruments, and differing neuroimaging techniques may partly explain the inconsistencies observed between studies, making it difficult to develop a cohesive neurocognitive model of EI.

### Translational implications of the neural basis of EI

EI has been closely linked to multiple social and health-related domains, including work, education, social relationships, mental health, neurodevelopmental disorders, and addictions. For example, EI is associated with psychological well-being. Higher EI correlates with greater life satisfaction and happiness, along with lower anxiety and burnout, independent of personality traits and intelligence (Di Fabio & Kenny, 2016; Fernández-Berrocal & Extremera, 2016; Llamas-Díaz et al., 2022; Miao, Humphrey & Qian, 2017). Similarly, higher EI is linked to a lower risk of major depressive disorder, fewer maladaptive behaviors, and reduced likelihood of suicide attempts (Domínguez-García & Fernández-Berrocal, 2018; Downey et al., 2008; Gómez-Leal et al., 2021; Okasha et al., 2023; Vega et al., 2022). EI is also significantly impaired in severe mental health conditions, including those within the schizophrenia-bipolar spectrum and borderline personality disorder, with these deficits being linked to adverse outcomes across the lifespan (Lewandowski, Pinkham & Van Rheenen, 2024; Norris-Nicholas, Malouff & Meynadier, 2025). In more specific domains, EI-based interventions have shown efficacy in improving emotional awareness, understanding, and regulation in individuals with Autism Spectrum Disorder (Thomson, Burnham Riosa & Weiss, 2015; Petrovska & Trajkovski, 2019; White et al., 2025), in reducing the likelihood of substance use (Kun & Demetrovics, 2010; Megías-Robles, Perea-Baena & Fernández-Berrocal, 2020), and in supporting the management of gaming disorder (Biolcati et al., 2025; Dang et al., 2019). A better understanding of the neural substrates of EI can be fundamental for advancing knowledge of these topics from a neuroscientific perspective and for informing practical applications.

## Conclusion

This systematic review provides substantial evidence supporting the view that EI is based on an interaction/integration of cognitive and emotional brain circuitry in conjunction with brain areas typically associated with social processing. Key brain regions implicated in EI included the insula, vmPFC, OFC, CC, amygdala, and superior longitudinal fasciculus. Brain networks involving these regions, such as the SMH circuitry, SCN, salience network, and DMN, were consistently highlighted in the reviewed literature. Furthermore, some studies supported the idea that the involvement of several of these brain regions in EI can be partially dissociated from cognitive intelligence. Other areas, including the dlPFC, cuneus, precuneus, fusiform gyrus, superior temporal gyrus, cerebellum, parahippocampal gyrus, inferior frontal gyrus, frontopolar gyrus, and superior parietal lobule, have also been reported; however, their specific role in EI needs to be further clarified. Moreover, additional research is required to more clearly differentiate between existing EI models at the neural level. Addressing the limitations of the current literature will help deepen our understanding of the brain mechanisms underlying EI, contributing to the development of a more detailed neural framework. This would constitute a significant step forward in advancing theoretical models of EI and could offer practical benefits for improving mental health and social-emotional well-being in the general population.

## Supplemental Information

10.7717/peerj.20539/supp-1 Supplemental Information 1 PRISMA checklist.

## Additional Information and Declarations

### Data Availability

The following information was supplied regarding data availability:

Raw data were not generated in this Systematic review/Meta-analysis.
